# A Novel Cæsarean Section

**Published:** 1912-04-06

**Authors:** H. Gordon Webb


### A NOVEL CÆSAREAN SECTION.

By H. GORDON WEBB, M.R.C.S., L.R.C.P.

Although no doubt such a proceeding as the following is fairly common in veterinary practice, it is not every day that a general practitioner is called upon at a moment's notice to perform a canine Cæsarean section.

I was attending a club patient in the North of England one day when my attention was drawn to a lovely specimen of a Yorkshire terrier, which was lying on its side on a couch apparently in extremis. The eyes were filming over and its poor attempts to wag its tail when spoken to were really pitiful to watch. Its time for parturition had passed about a week previously, and no puppy had been forthcoming. The condition evidently required urgent operative interference or the bitch was doomed. As the animal was a very valuable one I decided to do my best for it, relying on my scanty knowledge of canine anatomy derived from my necessary biological studies. However, I did not see any reason why a proceeding after the style of a complete Porro-cæsarean section should not answer. My wife is a fully trained surgical sister from London, and I left all necessary sterilising in her hands, while I turned a disused bedroom into an apology for an operating theatre. The table consisted of a marble-topped washstand with the back removed.

The bitch was anæsthetised in its mistress's arms and then fastened to the table by tying the two front paws to the legs of the table, and the two hind paws to the other legs of the table. The animal was thus fixed in position with its belly upwards. The district nurse continued the anæsthetic under my directions, as well that is to say as she could for amusement at the novelty of her patient.

Everything was then done as if the patient had been human, the skin being carefully sterilised and the necessary towels being placed around the area for operation.

I opened in the middle line, and out popped the head of a very large black fœtus, whose father must have been at least a retriever. I ligatured the soft tissues above and below the offending object and cut between ligatures both above and below. These stumps thus left after removal of the overcharged uterus were tied together by ligatures. The wound was closed in one layer. Gauze dressings were then applied and bandaged firmly on. This was further protected by a silicate bandage from chest to tail. Catheterisation was needed on the second day, but otherwise a perfectly uninterrupted recovery was made. The bandages were soaked off on the fourteenth day, and the bitch allowed to go about on her legs again. The wound had healed by first intention, and no sign of a hernia could be found at the end of eighteen months.

The motto, therefore, for a G.P. in the country who is forty miles from the nearest veterinary surgeon is to go on and do it, and trust to Providence to look after his patient if it happens not to be human.

